# Maternal Exercise during Pregnancy Impacts Motor Performance in 9-Year-Old Children: A Pilot Study

**DOI:** 10.3390/children10111797

**Published:** 2023-11-08

**Authors:** Nina Ferrari, Nikola Schmidt, Inga Bae-Gartz, Christina Vohlen, Miguel A Alejandre Alcazar, Konrad Brockmeier, Jörg Dötsch, Esther Mahabir, Christine Joisten

**Affiliations:** 1 Cologne Center for Prevention in Childhood, Youth/Heart Center Cologne, University Hospital of Cologne, 50937 Cologne, Germany; 2 Department for Physical Activity in Public Health, Institute of Movement and Neurosciences, German Sport University Cologne, 50933 Cologne, Germany; 3 Department for Pediatric Cardiology, Heart Center, University of Cologne, 50937 Cologne, Germany; 4 Department of Pediatrics and Adolescent Medicine, Faculty of Medicine, University of Cologne, 50931 Cologne, Germany; 5 Department of Pediatric and Adolescent Medicine, Translational Experimental Pediatrics-Experimental Pulmonology, Cologne Excellence Cluster on Stress Responses in Aging-Associated Diseases (CECAD), Faculty of Medicine and University Hospital Cologne, University of Cologne, 50937 Cologne, Germany; 6 The German Centre for Lung Research (DZL), Institute for Lung Health (ILH), Cardiopulmonary Institute (CPI), University of Giessen and Marburg Lung Centre (UGMLC), Justus-Liebig University Gießen, 35392 Gießen, Germany; 7 Comparative Medicine, Center for Molecular Medicine, Faculty of Medicine and University Hospital of Cologne, University of Cologne, 50931 Cologne, Germany

**Keywords:** motor skills, brain-derived neurotrophic factor, body mass index, lifestyle, sports intervention, exercise

## Abstract

The benefits of maternal physical activity during pregnancy are well documented, but long-term effects on the child have been less studied. Therefore, we conducted a pilot follow-up study of a lifestyle intervention during pregnancy that aimed to investigate whether exercise (endurance and strength training) during pregnancy affects motor performance and body composition of children up to 9 years of age, as well as possible influencing factors like brain-derived neurotrophic factor (BDNF) and lifestyle. Eleven mother−child pairs from the intervention and eight mother−child pairs from the control group were included. From birth up to 9 years of age, no differences in body mass index (BMI) or body mass index standard deviation scores (BMI-SDS) were found between the groups. Lifestyle intervention was one of the influencing factors for children’s cardiorespiratory endurance capacity and coordination. Moreover, maternal BDNF in the last trimester was significantly associated with running performance, which may be due to better neuronal development. This is the first study evaluating the effects of a lifestyle intervention during pregnancy on the motor performance 9 years after birth. Children’s participation in exercise programs over the past 9 years was not continuously recorded and therefore not included in the analysis. Even a cautious interpretation of these results indicates that a healthy lifestyle during pregnancy is essential in promoting child health. Larger studies and randomized control trials are necessary to confirm our results, especially those pertaining to the role of BDNF.

## 1. Introduction

Despite the myriad of reports on the influence of physical activity on maternal health [1], few have analyzed the effects of regular maternal physical activity on the resulting offspring. Regarding effects directly after birth, several surveys have concluded that exercise and regular physical activity during pregnancy reduce the risk of large newborns (macrosomia) [2], without affecting the odds of growth-restricted, small-for-gestational-age newborns, or preterm birth babies [2,3,4,5]. This is meaningful because, for example, macrosomic newborns have a higher risk of being overweight or obese at a later stage in life.

Preliminary studies have analyzed the short-term influences of exercise (mostly aerobic and endurance exercises) during pregnancy on children’s neurodevelopment [6,7,8,9], that is, 8–15 days to 24 months after delivery. Labonte-Lemoyne et al. [7] demonstrated that maternal moderate aerobe exercise during pregnancy had a beneficial effect on the neuroelectric response of the newborn brain. In addition, observational studies by Clapp et al. revealed a better performance in two out of six behavioral constellations 5 days after birth [10], but similar results in the Bailey scale (psychomotor scale and mental scale) at age 1 [6] among children whose mothers exercised during pregnancy. Regular moderate physical activity and aerobic exercise during pregnancy have also been linked to better neuromotor skills in 1-month-old female infants [9], with a lower risk of scoring in the abnormal range on certain aspects (e.g., fine motor skills) of the Ages and Stages Questionnaire (an infant development tool) at 1 year [11] and a slightly higher IQ in children at 48 months [12].

It remains unclear which factors or mechanisms affect children’s neurodevelopmental behavior. Brain-derived neurotrophic factor (BDNF), which seems to be a key molecule for brain development and neurogenesis, is considered one possibility. Animal studies have shown that fetuses and neonates of active mothers have higher BDNF concentrations in the hippocampus [13,14,15], which have been associated with improved memory and better spatial learning in young offspring [15]. It is controversial whether these effects are also found in other brain regions (e.g., cortex) [16,17]. Our own studies in a mouse model showed that regular moderate endurance exercise (voluntary wheel running) during pregnancy leads to increased BDNF serum levels at day 112 in the offspring of active mothers in contrast to the offspring of non-active mothers [18]. BDNF is known to cross the placental barrier [19], and pregnant physically active women tend to have higher BDNF levels in their blood at the end of pregnancy [18,20]. However, it is still unclear whether this also has a long-term effect on (neuro)motor development and performance in school-aged children. To date, very few studies have investigated the effects of exercise during pregnancy on children’s neurodevelopment or motor skills performance beyond the second year of life. Clapp et al. [21] analyzed the influence of regular moderate endurance exercise during pregnancy on oral language performance, intelligence, and motor skills performance in 5-year-old children. They found that the children of mothers who had exercised regularly during pregnancy exhibited higher general intelligence and better oral language skills. However, there were no differences in motor skills development [21] as assessed by the Bruininks-Oseretsky Test of Motor Proficiency. A recent study by Ellingsen et al. evaluated the effects of exercise during pregnancy on 7-year-old children using questionnaires [22] and, likewise, found no differences in motor skills between children whose mothers exercised during pregnancy and those who did not.

The aim of the present follow-up study was to investigate for the first time whether a multimodal lifestyle program (combination of endurance and strength training in addition to nutritional counselling) during pregnancy affects the motor performance of children up to 9 years of age, as well as which influencing factors determine motor performance. Both elements emphasize the importance of exercise during pregnancy.

## 2. Materials and Methods

The Ethics Committee of the German Sport University Cologne approved the study (ethics reference number: MAMA–10/7/2012 and MAMA PLUS–030/2022). The ethical principles of medical research involving human subjects were considered (Declaration of Helsinki). Prior to study entry, all study participants provided written informed consent confirming their voluntary participation.

### 2.1. Design of the Study

The MAMA (a Multimodal progrAm for the prevention of MAternal and fetal diseases) study [18] was a controlled trial conducted between 2012 and 2013 that evaluated how a multimodal lifestyle program including moderate endurance and strength exercise and healthy eating during pregnancy affected the mothers’ offspring. The study involved all women with singleton pregnancies up to the 13th week of gestation. Community-based gynecologists and obstetricians in the vicinity of Cologne helped in recruiting healthy pregnant women. Excluding criteria for participation in the study were defined as follows: women with hypertension, pre-existing diabetes mellitus, or other comorbidities that affect fetal growth, as well as non-German-speaking women. Fasting blood samples were collected at three time points: between weeks 13 and 14 (T1), weeks 23 and 24 (T2), and weeks 35 and 36 (T3) of gestation. The present study only considers the blood parameters of T3.

#### 2.1.1. Intervention during Pregnancy

The women in the intervention group participated in a supervised moderate endurance and strength exercise program from week 13 to at least week 36 of pregnancy. Each session consisted of at least 60–90 min twice a week. This program was based on the international guidelines for physical activity during pregnancy [23], as a combination of a moderate-intensity aerobic and strength-conditioning exercise is highly recommended during pregnancy.

At recruitment and subsequently throughout pregnancy, participants received at least three 90-min sessions of individual dietary counseling based on the recommendations of the German Health Information Service. The basis of the counseling was previously completed dietary recalls. Background information about the intervention design has been published elsewhere [18,24].

Women in the control group received routine prenatal care including information on diet and exercise from their gynecologist or obstetricians. They were neither encouraged nor discouraged from exercising or being active. Women in both the control and the intervention group underwent the same assessments.

#### 2.1.2. Follow-Up

**Figure 1:**
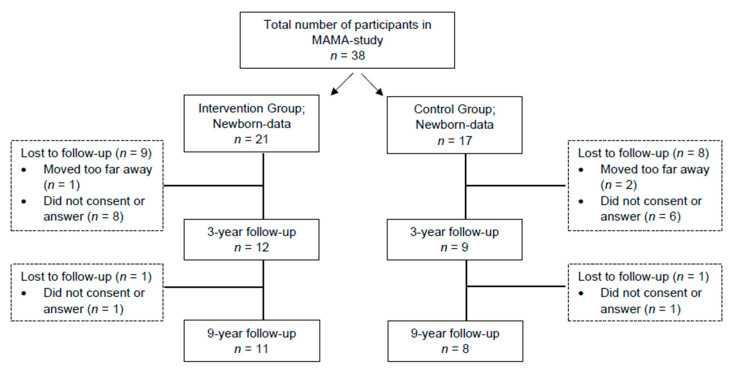
Diagram showing the number of pregnant women in the MAMA study and reasons for a lack of follow-up.

Based on the MAMA study described above, the anthropometry and motor performance of the children were examined again around 3 years (36–42 months) and 9 years after birth. For the first follow-up study at 3 years, 12 mother−child pairs from the intervention group and nine mother−child pairs from the control group were recruited. In the second follow-up study at 9 years, 11 mother−child pairs from the intervention group and 8 mother−child pairs from the control group participated (see Figure 1).

### 2.2. Anthropometry

#### 2.2.1. Baseline Maternal and Fetal Anthropometric Data

We determined the maternal body weight using a digital scale (Tanita Corp., Tokyo, Japan). Participants were dressed casually and were barefoot. We calculated maternal weight gain by the difference between the self-reported weight before pregnancy and the last weight recorded before delivery, and further classified excessive weight gain by the recommendations of the Institute of Medicine [25]. Maternal height was measured to the nearest 0.1 cm by using a metal stadiometer. From these values, the body mass index (BMI) from the mother was calculated in kg/m² and classified as follows: underweight (<18.5), normal weight (18.5–24.9), overweight (25–29.9), and obesity (≥30).

Furthermore, medical records or standardized questionnaires were used to obtain the following participant information: weight before pregnancy, status of parity, nationality, level of education, smoking status, physical activity, and mode of delivery.

We retrieved neonatal data from postnatal screening examinations: sex, birth weight, birth length, and Apgar score. Moreover, infants’ height and weight were obtained from examination records after 2, 3, 4, 5, and 7 years.

#### 2.2.2. Child Anthropometric Data at Follow-Up

At the first and the second follow-up, children’s height in meters (m) and weight in kilograms (kg) were assessed barefoot with a standardized stadiometer (Seca, Hamburg, Germany) and digital scale to the nearest 0.1 cm, as described elsewhere [26]. The children’s BMI in kg/m^2^ was calculated and classified as follows: BMI < 10th percentile for age and sex: underweight; BMI > 90th percentile for age and sex: overweight; BMI > 97th percentile for age and sex: obese [27]. Using the least mean squares (LMS) method for non-normally distributed characteristics [28], the BMI standard deviation score (SDS) was calculated

$${\mathrm{SDS}}_{\mathrm{LMS}}=\frac{{\left[\mathrm{BMI}/M[t]\right]}^{L[t]}-1}{L[t]S[t]}$$

M[t], L[t], and S[t] are variables describing the participant’s age and sex.

At the second follow-up, the body composition was measured using bioelectrical impedance analysis (BIA; Nutriguard-MS, Data Input GmbH, Pöcking, Germany). Fat mass and lean body mass were assessed to 0.1 kg and 0.1% with a four-point measurement (hand and foot). In accordance with the manufacturer’s instructions, the frequency of the measurement was 50 kHz [29]. During the measurement, the following parameters were determined: resistance (R), reactance (Xc), checksum (Σ), total resistance (Rtot.), and phase angle (φ). Fat and lean body mass (in kg) were analyzed using the NutriPlus program (NutriPlus, Data Input GmbH, Pöcking, Germany) [30].

Based on the following formula, the skeletal muscle mass (SMM) was calculated [31]:

SMM (kg) = [Ht²/R × 0.401) + (sex × 3.825) + (age × −0.071)] + 5.102

(Ht = height in cm; R = BIA − resistance in Ω; sex = 1 = male, 0 = female; age in years).

### 2.3. Laboratory Parameters

Fasting venous blood samples of the mother were collected (7.5 mL serum tube, S-Monovette, Sarstedt, Nümbrecht, Germany) in the morning and centrifuged at 4000 rpm for 10 min at 4 °C (Hettich Lab Technology, Tuttlingen, Germany). We pipetted the serum into new tubes for storage at −80 °C until analysis.

BDNF levels at 36 weeks of gestation were analyzed using a multiplex immunoassay kit (eBioscience, San Diego, CA, USA) and calculated with the Bio-Plex Manager 6.1 (Bio-Rad Laboratories, Hercules, CA, USA).

### 2.4. Motor Skills

Motor performance was assessed using the motor test battery for children aged 3 years (Kindergarten mobil; KiMo) [32] and the Dordel-Koch test (DKT) for children aged 6–16 years [33]. The KiMo test consists of five items: lateral jumping, standing long jump, sit-and-reach, one-leg stand, and shuttle−run test. The DKT test battery consists of seven test items: lateral jumping, standing long jump, sit-and-reach, sit-ups, one-leg stand, push-ups, and a 6 min run. Detailed information of the two test batteries is published elsewhere [32,33,34,35]. All test items can be classified according to gender and age.

The motor test battery was performed by trained personnel under standardized conditions. The KiMo test was performed with tight shoes, as recommended in the test manual. DKT was completed barefoot, as recommended in the test manual, except for the 6 min run.

### 2.5. Statistics, Data Management, and Data Analysis

All analyses were carried out in SPSS (IBM SPSS Statistics for Windows, version 29, IBM Corp., Armonk, NY, USA). We evaluated mean values and standard deviation for maternal and children’s anthropometric data and children’s motor skills data. Normal distribution was tested by the Kolmogorov−Smirnov test. Normally distributed parameters were analyzed with parametric tests. Otherwise, the Mann-Whitney U-test was used. The level of statistical significance was set to a *p*-value of <0.05, with associated 95% confidence intervals.

We examined two-group comparisons for the metric variables using the *t*-test. For the categorical and dichotomous variables, the χ^2^ and two-sided Fisher tests were used. Significant relationships among the data were determined by Pearson correlation (for normal distribution) or Spearman correlation (for non-normal distribution). Means of continuous variables with variance homogeneity were compared using analysis of variance (ANOVA). For variance heterogeneity, we used ANOVA with Welch correction.

To analyze the factors influencing motor performance (6 min run, lateral jumping, and standing long jump), backward multiple linear regression analysis was conducted.

The following parameters were included in the first model of the backward multiple linear regression analysis: group (0 = intervention, 1 = control), age of child (years), gender (0 = female, 1 = male), relative lean body mass of the child (percent), BMI-SDS of the child, and maternal BDNF at 36 weeks of gestation (picograms per milliliter). Independence of the residuals and multicollinearity were checked and respected.

## 3. Results

### 3.1. Mothers’ and Children’s Baseline Characteristics

The anthropometric, demographic, and obstetric data of the mother-child pairs who participated in the follow-up survey are presented in Table 1. Maternal characteristics at baseline were comparable between the intervention and control group. No differences were found in obstetric maternal and neonatal data (Table 1). Regarding leisure time physical activity during gestation, the participants in the control group were less physically active than those in the intervention group at baseline and at the end of pregnancy, but the differences were not significant due to some large outliers (baseline: 2.0 ± 1.3 vs. 4.1 ± 5.5 h/week, *p* = 0.307 using Mann-Whitney U-test; end: 3.7 ± 3.9 vs. 5.8 ± 3.9 h/week, *p* = 0.380 using Mann-Whitney U-test). However, during the intervention time (T2), significant differences were evident between the control group and the intervention group with respect to steps (7357.1 ± 2605.8 vs. 11,458.0 ± 2682.8 steps/day; *p* = 0.031 using Mann-Whitney U-test) and sedentary behavior (400.0 ± 147.6 vs. 203.1 ± 137.0 min/day; *p* = 0.038 using Mann-Whitney U-test). BDNF levels at the end of pregnancy were significantly higher in women from the intervention group compared with women in the control group (*p* = 0.022; see Table 1).

### 3.2. Anthropometry of Children at Follow-Up

Table 2 presents the children’s anthropometric data. Children from the intervention group were significantly older and therefore taller at the second follow-up. According to body weight, BMI, or BMI-SDS, no differences were found. The second follow-up survey also included a BIA, and no differences between the children from the intervention and the control groups were found (Table 2).

**Figure 2:**
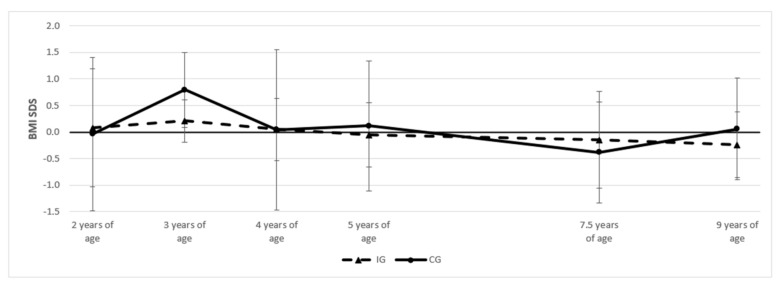
Trend of BMI-SDS; IG = intervention group, CG = control group.

The BMI-SDS trends in the children from ages 2 to 9 are presented in Figure 2. There were no significant differences between the intervention and the control groups at any measured time point.

### 3.3. Motor Performance

In the first follow-up, children from the control group performed better in the one-leg-stand test than children from the intervention group at the age of 3 (*p* = 0.024). There were no further significant differences between the groups, as shown in Table 3. Using the classification of test items and taking age and gender into account, no differences between the groups were found at the first follow-up.

The second follow-up 9 years after birth revealed that children in the intervention group were classified as very good, good, and satisfactory significantly more often than children in the control group in standing long jump (90.0% vs. 62.5%, *p* = 0.027). No further differences in the test items were found.

#### Factors Associated with Motor Performance at the Second Follow-Up

To analyze factors associated with cardiorespiratory and aerobic endurance capacity (6 min run in meters) a multiple linear regression analysis was used. The final model revealed that running performance was significantly influenced by age at the second follow-up (*β* = −1.865, *p ≤* 0.001), group (intervention: *β* = −1.689, *p ≤* 0.001), gender (female: *β* = –0.412, *p* = 0.052), and maternal BDNF in the third trimester (*β* = 0.290, *p* = 0.088). These results accounted for 80.6% of the variance. Table 4 presents detailed information.

To analyze the factors associated with agility and coordination (lateral jumping in counts), another multiple linear regression analysis was conducted. The end-model showed that the variable “intervention group” (*β* = –0.415, *p* = 0.098) explained 17.2% of the variance. Detailed information is presented in Table 5.

Using the standing long jump as a surrogate parameter for strength and power, we performed a multiple linear regression analysis with the standing long jump as the outcome variable. However, the backward analysis excluded all initial variables, and no final model was detected.

## 4. Discussion

In summary, we did not find any differences in body composition (BMI-SDS, fat, and muscle mass) between children who were born to mothers from the intervention group compared with children born to mothers from the control group 3 and 9 years after birth. However, participation in a supervised endurance and strength training exercise program during pregnancy, as well as BDNF level at 36 weeks of gestation, are influencing factors for motor performance in children.

Very few studies have analyzed the long-term effects of maternal exercise during pregnancy on the child thus far [12,21,22]. Regarding anthropometric data, there have been contradictory findings. Some authors have shown lower weight and less body fat after birth [36,37,38] or after 5 years [21] in children whose mothers were active during pregnancy. In contrast, a corresponding meta-analysis of 135 studies (*n* = 166,094 women) showed no link between exercise before and during pregnancy and infant outcomes or body composition (% body fat, body weight, and BMI) in the first years of life [2].

The hypothesis that an exercise program during pregnancy could influence the body composition of 9-year-olds is ambitious, as it assumes that the only difference between intervention and control groups is exercise during pregnancy, with later behavior being identical. In addition, maternal exercise would have to be the only factor influencing body composition. In our study, all mothers in both groups reported regular participation in sports activities for themselves and their children 9 years after delivery. All children were regularly active in school and sports clubs, regardless of whether they had been in the intervention or control group (100% vs. 87.5%, *p* = 0.250). This aspect may be more significant in influencing weight development and body composition than the exclusive consideration of the physical activity of the mother during gestation. Furthermore, no conclusions can be drawn regarding the paternal influence on the body composition of the child, as no specific data on the father were collected during pregnancy. However, it is known that not only the mother, but also the father, might influence the health and thus also body composition of the child via epigenetic influences [39].

It is noteworthy, however, that physical activity during pregnancy does affect children’s motor skills, although there are scant studies on the topic, and most employ questionnaire surveys rather than testing. Ellingsen et al. [22] evaluated the effect of an exercise program during pregnancy on neurodevelopment in 7-year-old children. In contrast with our study, they found that regular physical activity at a moderate intensity, during pregnancy did not affect motor skills in 7-year-old children. The study used the Five-to-Fifteen (FTF) questionnaire, which was answered by the parents, so no objective examination took place. In a recent study by Leao et al. [40], mother−infant pairs from the well-known randomized controlled PAMELA (Physical Activity for Mothers Enrolled in Longitudinal Analysis) study were evaluated 1, 2, and 4 years after delivery for language, cognition, and motor function. A modified version of the Battelle Development Inventory was used to measure motor function in the children at age 4. According to the authors, the assessment was conducted by trained interviewers, who were supervised by psychologists. Moreover, the assessment was based on direct observation of the children and interviews with caregivers [40]. However, there were no effects of regular physical activity and exercise during gestation in the motor domain of the children. In contrast with the studies mentioned above, we were able to confirm sporadic effects on motor function. This is remarkable, because motor performance of the children depends on many factors, such as physical activity in previous years. We did not continuously measure physical activity status or exercise performance over the last 9 years, which might have influenced our results. However, at the time of the motor performance assessment at the age of 9, there were no significant differences in the level of participation in exercise programs between children born to women in the intervention compared with children born to women in the control group (194.0 ± 106.9 min/week vs. 212.9 ± 148.2 min/week structured sports activities; *p =* 0.764).

In the multiple linear regression analysis, BDNF emerged along with age, group, and gender as a possible factor influencing motor function. Various studies have demonstrated that exercise can positively affect BDNF levels in the blood serum, even during pregnancy [18,20,41]. These elevated maternal BDNF levels may cross the placental barrier and thus could affect the offspring [42]. Animal studies have confirmed that maternal exercise during pregnancy also influences the offspring’s BDNF level in the long term [18]. There are also preliminary indications for the same in humans. Labonte-Lemoyne et al. [7] analyzed the influence of an exercise intervention during pregnancy on children’s brain activity a few days after delivery. It was shown that children born to physically active mothers had a smaller “slow positive mismatch response (SPMMR)” than children born to less physically active mothers [7]. According to the authors, an increase in the fetus’s supply of BDNF could explain the observed findings. However, long-term effects have not yet been studied in humans.

BDNF may be responsible for improved motor learning [43] and possibly also improved motor performance [44] due to multiple mechanisms that cannot be isolated. BDNF supports the growth and maintenance of neurons in the motor system and promotes the differentiation of progenitor cells into mature neurons. In addition, BDNF influences synaptic plasticity in the motor cortex and motor circuits [45], which enables the brain to adapt to new locomotor behaviors and facilitates motor learning. Furthermore, BDNF can influence the release of neurotransmitters such as glutamate and gamma-aminobutyric acid, which are involved in signal transmission between neurons [46,47]. This may improve communication within the motor circuits and thus lead to improved motor function. However, the extent to which these mechanisms were already positively influenced in the child by maternal exercise during pregnancy remains unclear.

### Strengths and Limitations

One strength of the present study is that the exercise program during pregnancy was supervised twice a week over a period of at least 20 weeks. Another strength lies in the objectively measured motor performance and standardized collection of anthropometric data. All values were collected simultaneously by trained staff. The study period of 9 years represents a further strength of the study, despite the fact that important determinants of motor performance (e.g., children’s physical activity status) could not be recorded continuously over this long period. However, this study was a controlled pilot study with a small number of participants, which might have influenced our results. As the sample size was small, the activity time at the end of pregnancy was no longer significantly different. This can be interpreted as a limitation. Nevertheless, women in the intervention group showed a significantly higher level of activity in the middle of pregnancy (T2) compared with the control group. Another limitation is the recruitment radius. The participants were all recruited via community-based obstetricians in the area around Cologne. Thus, only a selected collective (normal weight and high socio-economic status) was assessed. Therefore, the results may provide a first point of reference, but should be interpreted cautiously. Another limitation of this pilot study is that paternal aspects were not considered. It is already known that not only the mother, but also the father, can influence the health of the child via epigenetic influences [48]. In this context, Riyahi et al. [49] demonstrated in an animal study that paternal spatial training before conception increased BDNF in the hippocampus of the following generations (F1 and F2 male offspring).

## 5. Conclusions

Exercise during pregnancy may partially impact maternal BDNF levels and motor performance in children 9 years after birth. Apart from that, no differences in body composition were found.

Considering the existing limitations such as small sample sizes, non-continuous monitoring of determinants such as body weight, or exercise status, our results are meaningful because there have been very few studies examining the long-term effects of maternal physical activity on children’s health and/or motor performance. Therefore, our findings highlight the importance of a healthy lifestyle during pregnancy, despite the results being based on a pilot study. Further studies with a larger cohort and objectively measured motor performances are necessary to confirm our findings. Furthermore, it seems reasonable to also evaluate paternal aspects in further studies.

## Figures and Tables

**Table 1:** Anthropometric, demographic, and obstetric data of the mother-child pairs ^1^.

Parameter	Total Population Mean ± SD/%	Ig Mean ± SD/%	CG Mean ± SD/%	*p*-Value
**Baseline characteristics of the mother**				
Age (year)	30.9 ± 3.5 (*n* = 19)	31.4 ± 4.1 (*n* = 11)	30.2 ± 2.7 (*n* = 8)	0.484 ^+^
Height (m)	1.68 ± 0.05 (*n* = 19)	1.69 ± 0.06 (*n* = 11)	1.68 ± 0.05 (*n* = 8)	0.752 ^+^
Weight before pregnancy (kg)	63.6 ± 7.8 (*n* = 19)	62.5 ± 7.1 (*n* = 11)	65.1 ± 9.1 (*n* = 8)	0.516 ^+^
BMI before pregnancy (kg/m^2^)	22.2 ± 2.1 (*n* = 19)	21.8 ± 2.0 (*n* = 11)	22.9 ± 2.3 (*n* = 8)	0.308 ^+^
BMI classification before pregnancy				
Underweight	0.0% (*n* = 0)	0.0% (*n* = 0)	0.0% (*n* = 0)	0.080 ^§^
Normal weight	89.5% (*n* = 17)	100.0% (*n* = 11)	75.0% (*n* = 6)	
Overweight	10.5% (*n* = 2)	0.0% (*n* = 0)	25.0% (*n* = 2)	
Obese	0.0% (*n* = 0)	0.0% (*n* = 0)	0.0% (*n* = 0)	
Nationality (German)	94.7% (*n* = 18)	90.9% (*n* = 10)	100.0% (*n* = 8)	0.381 ^§^
Primipara	84.2% (*n* = 16)	90.9% (*n* = 10)	75.0% (*n* = 6)	0.348 ^§^
**Obstetric data of the mother**				
Weight gain during pregnancy (kg)	17.7 ± 6.5 (*n* = 18)	17.5 ± 5.0 (*n* = 11)	18.1 ± 8.9 (*n* = 7)	0.883 ^+^
Classification of weight gain (IOM)				0.429 ^§^
Below recommendation	11.2% (*n* = 2)	18.1% (*n* = 2)	0.0% (*n* = 0)	
Within recommendation	44.4% (*n* = 8)	36.4% (*n* = 4)	57.1% (*n* = 4)	
Above recommendation	44.4% (*n* = 8)	45.5% (*n* = 5)	42.9% (*n* = 3)	
Mode of delivery				
Vaginal delivery (normal)	49.9% (*n* = 9)	40.0% (*n*= 4)	62.5% (*n* = 5)	
Vaginal delivery (instrumental)	16.7% (*n* = 3)	20.0% (*n* = 2)	12.5% (*n* = 1)	0.813 ^§^
Caesarean section (elective)	16.7% (*n* = 3)	20.0% (*n* = 2)	12.5% (*n* = 1)	
Caesarean section (emergency)	16.7% (*n* = 3)	20.0% (*n* = 2)	12.5% (*n* = 1)	
**Maternal laboratory parameter at 36 weeks of gestation**				
BDNF	5175.4 ± 2698.9 (*n* = 18)	6284.6 ± 2468.5 (*n* = 11)	3432.4 ± 2164.0 (*n* = 7)	**0.022 ^+^**
**Data of the newborn**				
Female sex	63.2% (*n* = 12)	72.7% (*n* = 8)	50.0% (*n* = 4)	0.377 ^§^
Birth weight (g)	3482.6 ± 407.5 (*n* = 19)	3505.9 ± 338.1 (*n* = 11)	3450.6 ± 511.4 (*n* = 8)	0.795 ^+^
Birth length (cm)	51.7 ± 2.6 (*n* = 19)	51.9 ± 2.7 (*n* = 11)	51.4 ± 2.5 (*n* = 8)	0.665 ^+^
Classification of birth weight according to gestational age				
SGA (<10. Percentile)	0.0% (*n* = 0)	0.0% (*n* = 0)	0.0% (*n* = 0)	
Appropriate for gestational age (10–90 Percentile)	100.0% (*n* = 19)	100.0% (*n* = 11)	100.0% (*n* = 8)	-
LGA (>90 Percentile)	0.0% (*n* = 0)	0.0% (*n* = 0)	0.0% (*n* = 0)	

Data shown as means ± standard deviations or % (*n*). IG = intervention group; CG = control group; BMI = body mass index, IOM = Institute of Medicine; SGA = small for gestational age; LGA = large for gestational age. ^1^ Only those mother-child pairs were taken into account who also took part in the follow-up; ^+^ unpaired *t*-test, ^§^ chi-square test.

**Table 2:** Anthropometric data and body composition by bioelectrical impedance analysis of the children at 9 years follow-up (second follow-up).

Parameter	All	IG	CG	*p*-Value
Age (years) 2nd follow-up	9.4 ± 0.7 (*n* = 19)	9.9 ± 0.2 (*n* = 11)	8.7 ± 0.5 (*n* = 8)	**≤0.001 ^+^**
Height (meters) 2nd follow-up	1.38 ± 0.07 (*n* = 19)	1.41 ± 0.06 (*n* = 11)	1.33 ± 0.06 (*n* = 8)	**0.005 ^+^**
Weight (kg) 2nd follow-up	31.5 ± 5.1 (*n* = 19)	32.9 ± 4.3 (*n* = 11)	29.4 ± 5.7 (*n* = 8)	0.151 ^+^
BMI (kg/m^2^) 2nd follow-up	16.5 ± 1.7 (*n* = 19)	16.3 ± 1.4 (*n* = 11)	16.7 ± 2.0 (*n* = 8)	0.656 ^+^
BMI-SDS 2nd follow-up	−0.1 ± 0.8 (*n* = 19)	−0.2 ± 0.6 (*n* = 11)	0.06 ± 1.0 (*n* = 8)	0.422 ^+^
Fat mass (kg) 2nd follow-up	5.0 ± 2.4 (*n* = 18)	5.4 ± 2.0 (*n* = 11)	4.3 ± 2.9 (*n* = 7)	0.360 ^+^
Fat mass (%) 2nd follow-up	15.4 ± 6.0 (*n* = 18)	16.4 ± 5.2 (*n* = 11)	13.8 ± 7.2 (*n* = 7)	0.596 ^#^
Lean body mass (kg) 2nd follow-up	26.7 ± 3.7 (*n* = 18)	27.4 ± 3.5 (*n* = 11)	25.5 ± 3.9 (*n* = 7)	0.295 ^+^
Lean body mass (%) 2nd follow-up	84.6 ± 5.9 (*n* = 18)	83.5 ± 5.1 (*n* = 11)	86.2 ± 7.1 (*n* = 7)	0.367 ^+^
Skeletal muscle mass (kg) 2nd follow-up	17.2 ± 2.6 (*n* = 18)	17.2 ± 3.1 (*n* = 11)	17.1 ± 1.7 (*n* = 7)	0.937 ^+^
Skeletal muscle mass (%) 2nd follow-up	54.9 ± 9.0 (*n* = 18)	52.5 ± 7.6 (*n* = 11)	58.8 ± 10.3 (*n* = 8)	0.154 ^+^

Data shown as means ± standard deviations. IG = intervention group; CG = control group; ^+^ unpaired *t*-test; ^#^ Mann−Whitney U-Test.

**Table 3:** Motor performance of the children at 3 years (first follow-up) measured by KiMo test battery and 9 years follow-up (second follow-up) measured by DKT test battery.

Parameter	All	IG	CG	*p*-Value
Lateral jumping (counts) 1st follow-up	11.2 ± 7.7 (*n* = 19)	11.6 ± 8.2 (*n* = 12)	10.6 ± 7.3 (*n* = 7)	0.916 *
Sit and reach (cm) 1st follow-up	3.8 ± 3.9 (*n* = 19)	4.3 ± 4.1 (*n* = 12)	3.1 ± 3.4 (*n* = 7)	0.344 *
Standing long jump (cm) 1st follow-up	57.5 ± 9.4 (*n* = 19)	55.9 ± 9.4 (*n* = 12)	60.1 ± 9.5 (*n* = 7)	0.069 *
One leg stand (counts) 1st follow-up	22.2 ± 7.6 (*n* = 13)	25.6 ± 5.4 (*n* = 8)	16.6 ± 7.7 (*n* = 5)	0.024 *
Shuttle run test (seconds) 1st follow-up	14.0 ± 2.3 (*n* = 19)	14.2 ± 1.8 (*n* = 12)	13.6 ± 3.1 (*n* = 7)	0.456 *
Lateral jumping (counts) 2nd follow-up	60.0 ± 10.4 (*n* = 19)	62.3 ± 8.6 (*n* = 11)	56.9 ± 12.3 (*n* = 8)	0.436 *
Sit and reach (cm) 2nd follow-up	1.3 ± 7.4 (*n* = 19)	1.3 ± 8.1 (*n* = 11)	1.3 ± 6.9 (*n* = 8)	0.790 *
Standing long jump (cm) 2nd follow-up	138.4 ± 16.2 (*n* = 19)	138.2 ± 14.4 (*n* = 11)	138.6 ± 19.5 (*n* = 8)	0.164 *
Sit ups (counts) 2nd follow-up	16.3 ± 5.9 (*n* = 19)	17.8 ± 4.5 (*n* = 11)	14.3 ± 7.3 (*n* = 8)	0.834 *
One leg stand (counts) 2nd follow-up	0.8 ± 1.4 (*n* = 19)	0.5 ± 1.2 (*n* = 11)	1.1 ± 1.6 (*n* = 8)	0.387 ^#^
Push-ups (counts) 2nd follow-up	4.3 ± 4.0 (*n* = 19)	4.1 ± 2.9 (*n* = 11)	4.7 ± 5.4 (*n* = 8)	0.735 ^#^
6-min run (meters) 2nd follow-up	1142.8 ± 111.1 (*n* = 17)	1165.2 ± 106.0 (*n* = 11)	1101.8 ± 117.9 (*n* = 6)	0.198 *

Data are shown as means ± standard deviations; IG = intervention group; CG = control group; * univariate variance analysis adjusted for age and BMI; ^#^ Welch-ANOVA.

**Table 4:** Backward multiple linear regression analysis of the 6 min run (first and final models presented).

Model		*β*-Coefficient	*p*-Value	R^2^
1	Age of child [year]	−1.757	0.002	0.833
	Group	−1.546	0.004	
	Gender	−0.470	0.049	
	BMI-SDS of child	−0.088	0.578	
	Lean body mass of child [%]	0.155	0.377	
	Maternal BDNF at 36 weeks of gestation [pg/mL]	0.294	0.104	
3	Age of child [year]	−1.865	≤0.001	0.806
	Group	−1.689	≤0.001	
	Gender	−0.412	0.052	
	Maternal BDNF at 36 weeks of gestation [pg/mL]	0.290	0.088	

BDNF = brain-derived neurotrophic factor.

**Table 5:** Backward multiple linear regression analysis of lateral jumping (first and final models presented).

Model		*β*-Coefficient	*p*-Value	R^2^
**1**	Age of child [year]	0.031	0.970	0.246
	Group	−0.451	0.563	
	Gender	0.105	0.740	
	BMI-SDS of child	−0.030	0.909	
	Lean body mass of child [%]	−0.232	0.507	
	Maternal BDNF at 36 weeks of gestation [pg/mL]	−0.207	0.548	
6	Group	−0.415	0.098	0.172

BDNF = brain-derived neurotrophic factor.

## Data Availability

Data are contained within the article.
